# Effects of the COVID-19 Pandemic on Hand and Arm Dysfunction: A Google Trends Analysis

**DOI:** 10.7759/cureus.62369

**Published:** 2024-06-14

**Authors:** Jasmin Valenti, Lainey G Bukowiec, Peter Rhee

**Affiliations:** 1 School of Medicine, Hackensack University Medical Center, Nutley, USA; 2 Orthopedic Surgery, Mayo Clinic, Rochester, USA

**Keywords:** hand pain, de quervain tenosynovitis, carpal tunnel, arm, hand

## Abstract

Introduction

The COVID-19 pandemic prompted individuals to make a number of lifestyle alterations. Few studies have examined the development of any hand and/or arm dysfunctions that may have resulted. The purpose of this study was to identify hand and/or arm overuse injuries that may have occurred as a result of the stay-at-home orders during the COVID-19 pandemic.

Methods

A Google Trends analysis of the terms “hand pain,” “carpal tunnel syndrome,” “cubital tunnel syndrome,” “trigger finger,” “de Quervain tenosynovitis,” “elbow pain,” “tennis elbow,” “golfer’s elbow,” “thumb base arthritis,” and “extensor carpi ulnaris tenosynovitis” in the United States, United Kingdom, Canada, and India was performed from June 2019 to January 2023. The noted timeframe was divided into quarters of 47 weeks, with the first quarter (June 2, 2019, through April 19, 2020) serving as a pre-pandemic baseline. The analysis compared initial results noted in the first quarter to individual results from the second, third, and fourth quarters.

Results

The most notable findings were the upward trends of the terms “hand pain,” “carpal tunnel,” and “trigger finger.” Specifically, India showed a significant increase in the terms “hand pain” and “carpal tunnel syndrome” in the second, third, and fourth quarters. The United States additionally showed a significant upward trend in the terms “carpal tunnel syndrome” and “trigger finger” in the second, third, and fourth quarters. The United Kingdom also reported a significant upward trend in the term “trigger finger” in the second, third, and fourth quarters.

Conclusion

Numerous factors likely contributed to the increased interest in these terms, such as the increase in telework and associated mobile device usage due to lockdown during the COVID-19 pandemic. Movements associated with performing these tasks may have led to an increased prevalence of hand pain, thus prompting increased queries of these terms through an online search engine.

## Introduction

The COVID-19 pandemic had profound implications on the working environment of individuals all over the world. The pandemic was officially declared by the World Health Organization (WHO) on March 11, 2020, leading to lockdowns in many countries [1]. Individuals and companies were forced to make changes in their lifestyles to accommodate various socio-political restrictions. One of the most notable changes that occurred was the rise in telework, with many countries, such as Great Britain, having nearly half of their workforce working from home by April 2020 [2].

A number of studies have examined the health effects of increased telework during the pandemic, with many reporting negative impacts on physical and mental well-being [3,4]. Specific to the musculoskeletal system, there has been an increased number of patients seeking medical evaluation for musculoskeletal issues, especially low back and neck pain [5]. However, only a few studies have examined whether the rise of telework and other increased activities during the pandemic has been associated with an increased prevalence of hand and arm dysfunction and pain [3,6].

Some of these lifestyle changes remain relevant even in the post-pandemic landscape. Specifically, telework will likely remain an integral part of the workforce and many individuals will prefer to continue some form of telework in the future [7]. Similarly, the usage of mobile devices for telework and social media was noted to have had a marked increase during and after the public health emergency. Therefore, this study aims to examine the potential association between the rise of telework and other tasks (i.e., miscellaneous mobile device usage) during the COVID-19 pandemic and the trends in internet search engine utilization for topics related to elbow, hand, and wrist pathologies.

## Materials and methods

Data source

Google Trends is an application that tracks weekly trends of individual search terms queried on Google’s search engine (Alphabet Inc., Mountain View, United States). The data provided by Google Trends is publicly available and de-identified [8]. Institutional review board approval was therefore not obtained to conduct this analysis. Terms are arranged by specific words or phrases and given a value in terms of “interest over time.” Interest over time is measured on a scale of 0 to 100.

A value of 0 indicates a very low search volume when compared to the overall search volume of a selected region and time frame. However, this does not necessarily mean that there were no searches for the selected term. A value of 50 means the term was proportionally half as popular relative to its internet traffic during the all-time peak popularity. A value of 100 represents the “peak popularity” for the term, meaning when the term was most highly searched in comparison to all other Google searches within a given region and time frame.

Google Trends normalizes its data, meaning interest over time is in reference to the proportion of all searches on Google for a selected time and location. Each data point is divided by the total searches within the selected region and time frame to compare relative popularity. This is to prevent areas with high population density and thus higher search volumes from scoring disproportionately high on the scale. This method allows researchers to make comparisons of a term’s popularity between different regions, while accounting for differences in population density, within a set time frame.

In this study, Google Trends was used to compare mean interest over time within selected regions across a 3.5-year time span. Using this methodology poses some limitations, the most obvious being that a large increase or decrease in overall search volume will influence a term’s interest over time value. For example, a major news event in one of the selected areas may cause a spike in the overall search volume on Google, leading to a decrease in the interest over time value of a selected term [8]. In addition, Google Trends does not provide the exact quantitative amount of queries, limiting data analysis. Instead, Google Trends uses a sample of all Google searches for a selected time and region.

Analysis 

The purpose of this study was to evaluate changes in the interest of elbow, hand, and wrist conditions via Google trends before, during, and after the COVID-19 pandemic. A trend analysis of the terms “hand pain,” “carpal tunnel syndrome,” “cubital tunnel syndrome,” “trigger finger,” “de Quervain tenosynovitis,” “elbow pain,” “tennis elbow,” “golfer’s elbow,” “thumb base arthritis,” and “extensor carpi ulnaris tenosynovitis” was performed to examine if there were any changes in the interest over time during the COVID-19 pandemic.

Query terms were organized by the search term, the related diagnosis, and whether or not viable data was available (Table 1). A term was considered to be non-viable if Google Trends could not generate a trend analysis in one of the selected regions of the study due to a lack of data. All queries were performed on January 10, 2023. All query categories available on the Google Trends site were included as part of the search (i.e., Finance, Health, Shopping, etc.). Weekly trends were analyzed beginning June 1, 2019, to January 01, 2023. The start date of June 1, 2019, was selected to obtain a sample of search trends prior to the COVID-19 pandemic. The time frame selected spans a total of 188 weeks. All search terms within the above time frame were analyzed in four selected regions with a high population of English language speakers such as the United States, the United Kingdom, Canada, and India.

**Table 1:** Utilized terms with associated orthopedic conditions ECU: extensor carpi ulnaris

Term used	Orthopedic condition	Viable data
Hand pain	Not applicable	Yes
Carpal tunnel syndrome	Carpal tunnel syndrome	Yes
Cubital tunnel syndrome	Cubital tunnel syndrome	Yes
Trigger finger	Trigger finger	Yes
De Quervain tenosynovitis	De Quervain tenosynovitis	Yes
Elbow pain	Not applicable	Yes
Tennis elbow	Lateral epicondylitis	Yes
Golfer’s elbow	Medial epicondylitis	Yes
Thumb base arthritis	Thumb base arthritis	No
ECU tenosynovitis	ECU tenosynovitis	No

Regression models for all viable terms were created using Excel to investigate trends among the four regions selected. All 188 weekly interest over time values were included in the models. Overall trends were then further analyzed by dividing the 188-week timeframe into 47-week quarters. The first quarter consists of the time period June 2, 2019, through April 19, 2020; the second quarter consists of the time frame April 26, 2020, through March 14, 2021; the third quarter consists of the time frame March 21, 2021, through February 6, 2022; the fourth quarter consists of the time frame February 13, 2020, through January 1, 2023.

The first quarter data (baseline) was compared by Student’s paired t-test in a pair-wise approach to the second, third, and fourth quarters separately. All tests were one-sided and statistical significance was set to a p-value of < 0.05.

A sub-analysis of the term “carpal tunnel syndrome” was performed using four selected states within the United States: New York, California, Florida, and Texas to analyze how different stay-at-home policies by state may have impacted the condition. The “carpal tunnel syndrome” term alone was selected as it had the most statistically significant differences over time in the national comparison.

## Results

Hand pain

**Figure 1:**
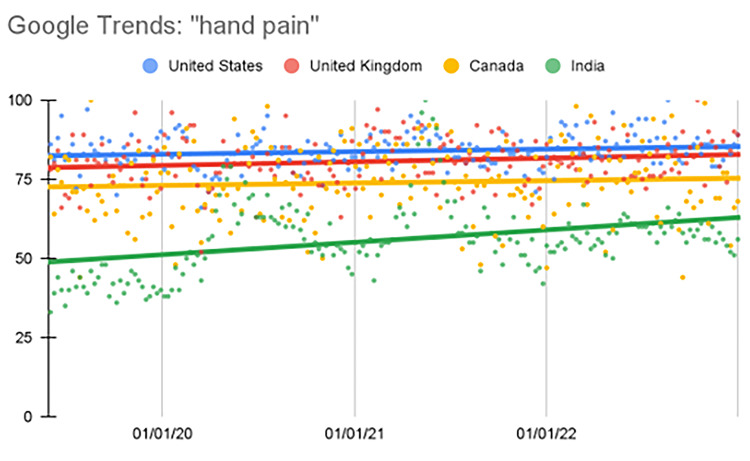
Regression model for "hand pain" The X-axis covers the period from June 2, 2019, through January 1, 2023. The Y-axis represents the “interest over time” for the term on a scale of 0-100, with 100 representing the maximum “interest over time” value for the selected region.

The United States showed a significant increase in this search term during the fourth quarter relative to the first quarter prior to the pandemic (p=0.004). In India, the second, third, and fourth quarters showed a significant increase in the search term (p<0.001) (Table 2, Figure 1). 

**Table 2:** Relative change per quarter "hand pain" Comparison of interest over time (searches per time) values per quarter. All statistical analyses, including relative change, were calculated using the “first quarter” as a baseline value. ^*^Represents a p-value < 0.05 when compared to the “first quarter” value

	06/02/19-04/19/20	04/26/20-03/14/21	03/21/21-02/06/22	02/13/22-01/01/23
	First quarter	Second quarter	Third quarter	Fourth quarter
United States	82.53	83.23 (0.85%)	83.87 (1.62%)	85.79 (3.94%)^*^
United Kingdom	79.06	80.09 (1.29%)	81.87 (3.55%)	81.89 (3.58%)
Canada	71.28	75.15 (5.43%)	74.13 (4.00%)	75.19 (5.49%)
India	44.04	59.36 (34.78%)^*^	62.17 (41.16%)^*^	58.02 (31.74%)^*^

Carpal tunnel syndrome (national comparison)

**Figure 2:**
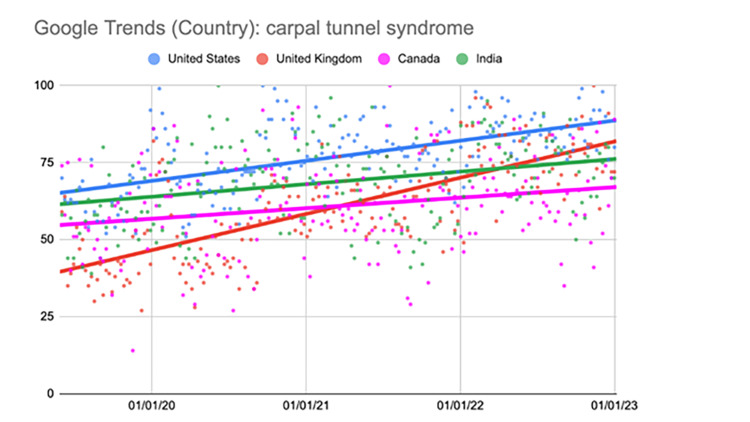
Regression model for "carpal tunnel syndrome" (national comparison) The X-axis covers the period from June 2, 2019, to January 1, 2023. The Y-axis represents the “interest over time” for the term on a scale of 0-100, with 100 representing the maximum “interest over time” of the selected region.

In the United States, the second, third, and fourth quarters showed a significant increase in this search term (p<0.001). The United Kingdom displayed a significant increase in the second quarter (p=0.002). This trend further continued in the third and fourth quarters (p<0.001). Canada did not show a significant increase until the fourth quarter (p=0.006). India experienced a significant increase in the second, third, and fourth quarters. During the second and fourth quarters, there was a significant increase (p<0.001). During the third quarter, there was also a significant increase but to a slightly lesser extent (p=0.02) (Table 3, Figure 2).

**Table 3:** Relative change per quarter "carpal tunnel syndrome" (national comparison) Comparison of interest over time (searches per time) values per quarter. All statistical analyses, including relative change, were calculated using the “first quarter” as a baseline value. ^*^Represents a p-value < 0.05 when compared to the “first quarter” value

	06/02/19-04/19/20	04/26/20-03/14/21	03/21/21-02/06/22	02/13/22-01/01/23
	First quarter	Second quarter	Third quarter	Fourth quarter
United States	66.74	75.85 (13.64%)^*^	80.02 (19.89%)^*^	85.26 (27.73%)^*^
United Kingdom	46.09	54.17 (17.54%)^*^	65.47 (42.06%)^*^	77.49 (68.14%)^*^
Canada	57.17	59.98 (4.91%)	60.15 (5.21%)	66.38 (16.11%)^*^
India	59	75.40 (27.80%)^*^	65.09 (10.31%)^*^	75.96 (28.74%)^*^

Carpal tunnel syndrome (state comparison)

**Figure 3:**
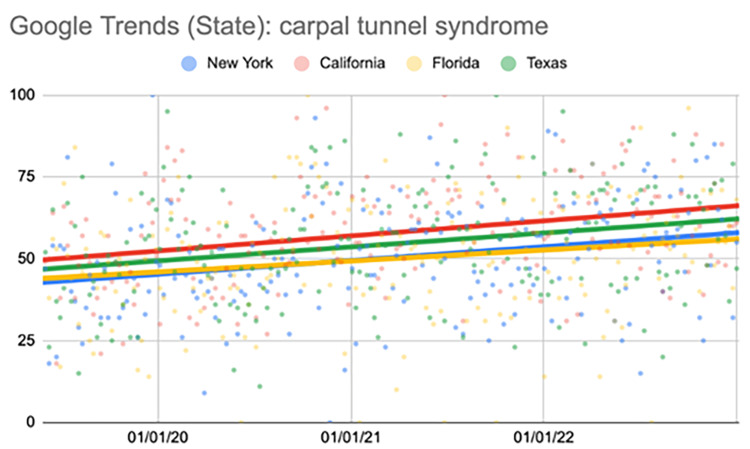
Regression model for "carpal tunnel syndrome" (state comparison) The X-axis covers the period from June 2, 2019, to January 1, 2023. The Y-axis represents the “interest over time” for the term on a scale of 0-100, with 100 representing the maximum “interest over time” value for the selected region.

New York showed a significant increase in the search term during the third (p=0.02) and fourth quarters (p<0.001). California showed a significant increase during the third and fourth quarters (p<0.001). Florida showed a significant increase during the fourth quarter (p=0.01). Texas showed a significant increase during the third quarter (p=0.04) and fourth quarters (p=0.01) (Table 4, Figure 3). 

**Table 4:** Relative change per quarter "carpal tunnel syndrome" (state comparison) Comparison of interest over time (searches per time) values per quarter. All statistical analyses, including relative change, were calculated using the “first quarter” as a baseline value. ^*^Represents a p-value < 0.05 when compared to the “first quarter” value

	06/02/19-04/19/20	04/26/20-03/14/21	03/21/21-02/06/22	02/13/22-01/01/23
	First quarter	Second quarter	Third quarter	Fourth quarter
New York	44.55	48.32 (8.45%)	52.55 (17.96%)^*^	56.06 (25.84%)^*^
California	50.40	55.98 (11.06%)	63.43 (25.83%)^*^	62.04 (23.09%)^*^
Florida	44.79	52.19 (16.53%)	48.68 (8.69%)	54.47 (21.62%)^*^
Texas	49.96	50.96 (2.00%)	57.66 (15.42%)^*^	59.49 (19.08%)^*^

Cubital tunnel syndrome

**Figure 4:**
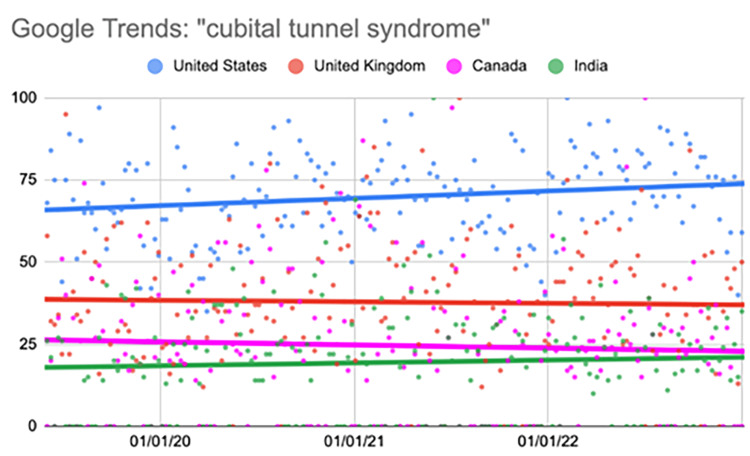
Regression model for “cubital tunnel syndrome” The X-axis covers the time period from June 2, 2019, to January 1, 2023. The Y-axis represents the “interest over time” for the term on a scale of 0-100, with 100 representing the maximum “interest over time” of the selected region.

The United States showed a statistically significant increase in the search term in the second (p=0.02) and fourth quarters (p<0.001) (Table 5, Figure 4).

**Table 5:** Relative change per quarter "cubital tunnel syndrome" Comparison of interest over time (searches per time) values per quarter. All statistical analyses, including relative change, were calculated using the “first quarter” as a baseline value. ^*^Represents a p-value < 0.05 when compared to the “first quarter” value

	06/02/19-04/19/20	04/26/20-03/14/21	03/21/21-02/06/22	02/13/22-01/01/23
	First quarter	Second quarter	Third quarter	Fourth quarter
United States	64.77	71.83 (10.91%)^*^	67.81 (4.70%)	75.23 (16.16%)^*^
United Kingdom	35.60	43.09 (21.04%)	35.11 (-1.37%)	37.66 (5.80%)
Canada	22.87	30.00 (31.16%)	21.30 (-6.88%)	24.32 (6.33%)
India	16.13	21.91 (35.88%)	20.30 (25.86%)	19.74 (22.43%)

Trigger finger

**Figure 5:**
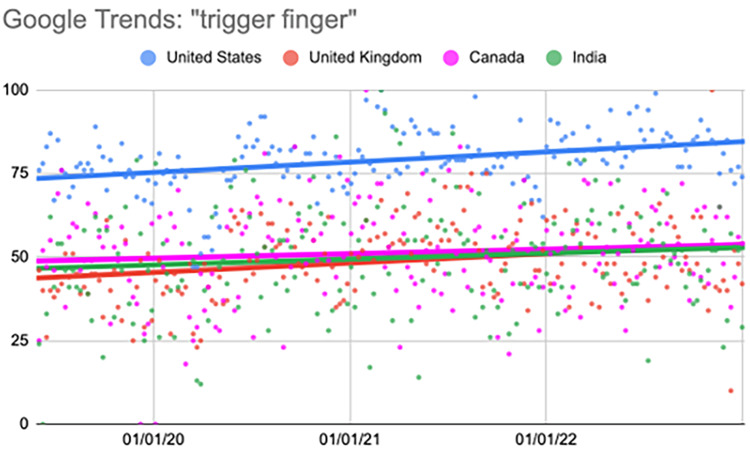
Regression model for "trigger finger" The X-axis covers the time period from June 2, 2019, to January 1, 2023. The Y-axis represents the “interest over time” for the term on a scale of 0-100, with 100 representing the maximum “interest over time” of the selected region.

The United States displayed a significant increase in the second quarter (p=0.003). This trend further continued in the third and fourth quarters (p<0.001). In the United Kingdom, the second, third, and fourth quarters showed a significant increase in the search term (p<0.001). Canada showed a significant increase during the second (p=0.005) and fourth quarters (p=0.03). India showed a significant increase during the second (p=0.008) and fourth quarters (p=0.009) (Table 6, Figure 5).

**Table 6:** Relative change per quarter "trigger finger" Comparison of interest over time (searches per time) values per quarter. All statistical analyses, including relative change, were calculated using the “first quarter” as a baseline value. ^*^Represents a p-value < 0.05 when compared to the “first quarter” value

	06/02/19-04/19/20	04/26/20-03/14/21	03/21/21-02/06/22	02/13/22-01/01/23
	First quarter	Second quarter	Third quarter	Fourth quarter
United States	72.70	79.47 (9.31%)^*^	80.55 (10.80%)^*^	83.51 (14.87%)^*^
United Kingdom	40.40	50.94 (26.07%)^*^	54.91 (35.91%)^*^	48.96 (21.17%)^*^
Canada	46.36	56.70 (22.30%)^*^	49.17 (6.06%)	53.00 (14.32%)^*^
India	43.26	53.60 (23.91%)^*^	50.11 (15.84%)	52.09 (20.41%)^*^

De Quervain tenosynovitis

**Figure 6:**
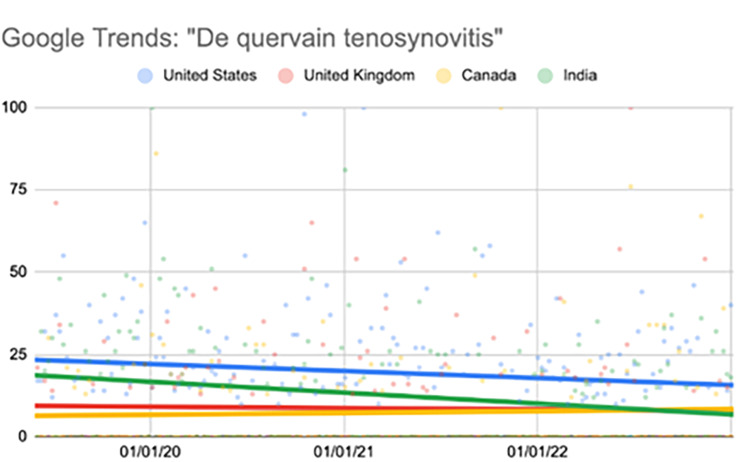
Regression model for "de Quervain tenosynovitis" The X-axis covers the time period from June 2, 2019, to January 1, 2023. The Y-axis represents the “interest over time” for the term on a scale of 0-100, with 100 representing the maximum “interest over time” of the selected region.

The United States showed a significant decrease in the search term during the fourth quarter (p=0.04). India showed a significant decrease during the second (p=0.03), third (p<0.001), and fourth quarters (p=0.01) (Table 7, Figure 6).

**Table 7:** Relative change per quarter "de Quervain tenosynovitis" Comparison of interest over time (searches per time) values per quarter. All statistical analyses, including relative change, were calculated using the “first quarter” as a baseline value. ^*^Represents a p-value < 0.05 when compared to the “first quarter” value

	06/02/19-04/19/20	04/26/20-03/14/21	03/21/21-02/06/22	02/13/22-01/01/23
	First quarter	Second quarter	Third quarter	Fourth quarter
United States	22.26	20.62 (-7.63%)	19.34 (-13.10%)	15.98 (-28.20%)^*^
United Kingdom	8.94	9.23 (3.33%)	7.06 (-20.95%)	9.32 (4.29%)
Canada	8.11	6.60 (-18.64%)	4.91 (-39.37%)	9.96 (22.83%)
India	21.02	11.38 (-45.85%)^*^	7.23 (-65.59%)^*^	11.23 (-46.38%)^*^

Elbow pain

**Figure 7:**
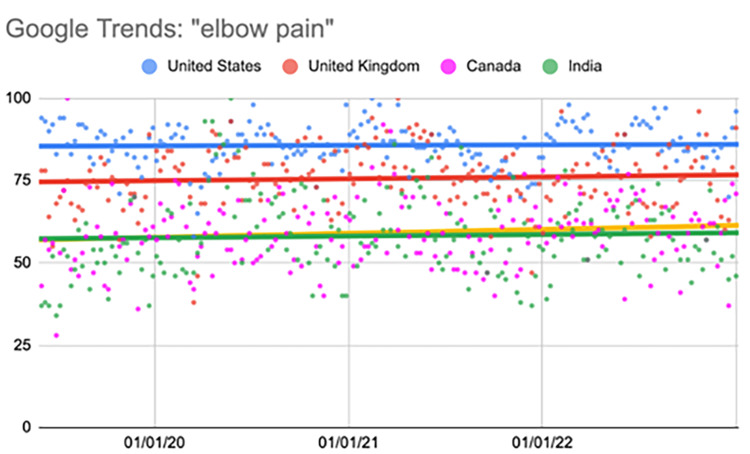
Regression model for “elbow pain” The X-axis covers the time period from June 2, 2019, to January 1, 2023. The Y-axis represents the “interest over time” for the term on a scale of 0-100, with 100 representing the maximum “interest over time” of the selected region.

The United States showed a significant increase during the fourth quarter (p=0.03). The United Kingdom showed a significant increase during the second (p=0.001) and fourth quarters (p=0.03). India showed a significant increase during the second quarter (p=0.001) (Table 8, Figure 7).

**Table 8:** Relative change per quarter "elbow pain" Comparison of interest over time (searches per time) values per quarter. All statistical analyses, including relative change, were calculated using the “first quarter” as a baseline value. ^*^Represents a p-value < 0.05 when compared to the “first quarter” value

	06/02/19-04/19/20	04/26/20-03/14/21	03/21/21-02/06/22	02/13/22-01/01/23
	First quarter	Second quarter	Third quarter	Fourth quarter
United States	84.68	87.15 (2.91%)	83.57 (-1.31%)	87.55 (3.39%)^*^
United Kingdom	72.23	79.02 (9.40%)^*^	75.72 (4.83%)	75.74 (4.86%)^*^
Canada	57.34	59.28 (3.38%)	59.26 (3.34%)	61.04 (6.46%)
India	53.21	63.21 (18.79%)^*^	58.26 (9.48%)	58.32 (9.60%)^*^

Tennis elbow

**Figure 8:**
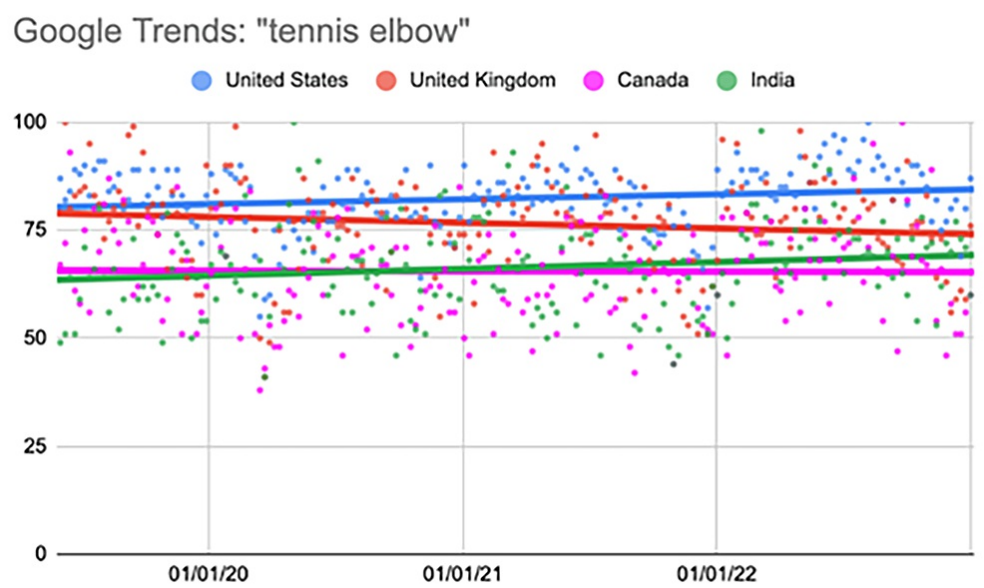
Regression model for “tennis elbow” The X-axis covers the time period from June 2, 2019, to January 1, 2023. The Y-axis represents the “interest over time” for the term on a scale of 0-100, with 100 representing the maximum “interest over time” of the selected region.

The United States showed a significant increase during the fourth quarter (p<0.001). India showed a significant increase during the second (p=0.004) and fourth quarters (p<0.001) (Table 9, Figure 8).

**Table 9:** Relative change per quarter "tennis elbow" Comparison of interest over time (searches per time) values per quarter. All statistical analyses, including relative change, were calculated using the “first quarter” as a baseline value. ^*^Represents a p-value < 0.05 when compared to the “first quarter” value

	06/02/19-04/19/20	04/26/20-03/14/21	03/21/21-02/06/22	02/13/22-01/01/23
	First quarter	Second quarter	Third quarter	Fourth quarter
United States	81.49	80.30 (-1.46%)	81.43 (-0.08%)	86.51 (6.16%)^*^
United Kingdom	77.96	75.40 (-3.28%)	76.36 (-2.05%)	76.26 (-2.18%)
Canada	66.45	64.04 (-3.62%)	63.26 (-4.80%)	68.30 (2.79%)
India	62.94	69.68 (10.72%)^*^	61.40 (-2.43%)	71.62 (13.79%)^*^

Golfer's elbow

**Figure 9:**
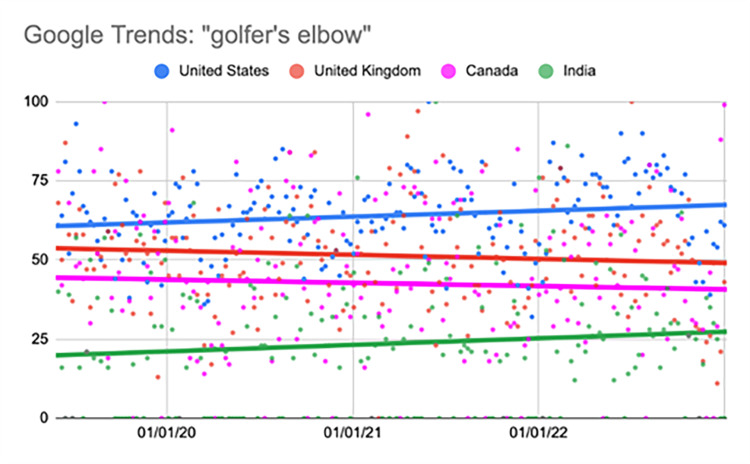
Regression model for “golfer’s elbow” The X-axis covers the time period from June 2, 2019, to January 1, 2023. The Y-axis represents the “interest over time” for the term on a scale of 0-100, with 100 representing the maximum “interest over time” of the selected region.

The United States showed a significant increase during the fourth quarter (p<0.001) (Table 10, Figure 9).

**Table 10:** Relative change per quarter "golfer's elbow" Comparison of interest over time (searches per time) values per quarter. All statistical analyses, including relative change, were calculated using the “first quarter” as a baseline value. ^*^Represents a p-value < 0.05 when compared to the “first quarter” value

	06/02/19-04/19/20	04/26/20-03/14/21	03/21/21-02/06/22	02/13/22-01/01/23
	First quarter	Second quarter	Third quarter	Fourth quarter
United States	60.38	63.06 (4.44%)	63.70 (5.50%)	69.17 (14.55%)^*^
United Kingdom	52.06	49.96 (-4.05%)	41.30 (1.55%)	42.70 (-2.78%)
Canada	42.26	44.06 (4.28%)	41.30 (-2.27%)	42.70 (1.06%)
India	20.36	22.68 (11.39%)	24.47 (20.17%)	26.98 (32.50%)

## Discussion

The current study examined search trends for a variety of elbow, hand, and wrist conditions through the Google search engine within the United States, United Kingdom, Canada, and India before and during the COVID-19 pandemic. These countries were specifically selected due to their high volume of English speakers, which matches the language of the selected search terms used for this study. The United States, United Kingdom, and Canada recognize English as their official language. India, despite Hindi being the most popular language within the country, was chosen as it has the second highest number of English speakers in the world behind the United States. The relative change between the mean of the first quarter, pre-pandemic or baseline, compared to the second, third, and fourth quarters were calculated to compare the relative interest of terms among different time periods throughout the COVID-19 pandemic.

The most dramatic finding is the positive trend in the respective population’s interest in the terms “hand pain,” “carpal tunnel,” and “trigger finger.” Results showed a significant increase in the search term “hand pain” in India throughout the second, third, and fourth quarters, ranging from a 31-41% relative increase in comparison with the first quarter. This correlates with an increase in the search term “carpal tunnel” in India throughout the second, third, and fourth quarters, along with an increase in the search term “trigger finger” during the second and fourth quarters. Similar findings in regards to “carpal tunnel” and “trigger finger” were also seen in the United States and the United Kingdom, which raised the question of what factors within these countries drove the changes in search trends.

Increased telework or increased time performing recreational or household activities during the pandemic may have contributed to the increased interest over time noted for carpal tunnel syndrome (CTS) and trigger finger. The population in India experienced a notable increase in the utilization of telework during the COVID-19 pandemic, which may have led to an overall increase in wrist movement within the population, leading to the increased search for hand pain and other hand conditions. In addition, the United States, Canada, and the United Kingdom also experienced an increase in telework utilization but to a lesser extent [9-11].

The influence of telework on orthopedic hand conditions 

Prior to India’s lockdown period during COVID-19, less than 20% of the population engaged in full-time telework. COVID-19 restrictions led to a dramatic increase in full-time telework to 70% between the periods of March 2020 and May 2020 [12]. Initially, it was thought that increased wrist movement associated with computer mouse usage may have contributed to the rising trends noted in India. However, literature regarding the increased usage of a desktop mouse and the development of CTS is controversial. Some sources cite excessive mouse usage as a risk factor for the development of CTS [13]. Yet recent meta-analyses have concluded that it is not possible to establish a direct causation [14]. Although many workplaces have transitioned to ergonomic devices, these have been shown to be overall ineffective in reducing pressure within the carpal tunnel, making the alteration of mouse and keyboard devices a poor line of prevention regarding the development and exacerbation of CTS [15].

Addressing the usage of other devices used to perform telework, specifically handheld devices (i.e., mobile phones, tablets), seems to be more promising [16,17]. Analyzing excessive usage of these devices is relevant, as companies have started to increasingly rely on employees to use their own personal devices for work, with mobile devices becoming necessary to perform many required tasks [18]. The shift to increased usage of handheld devices within the workplace led to a marked increase in time individuals spent using handheld devices during lockdown [19].

Excessive usage of smartphones has been associated with the development of CTS and de Quervain tenosynovitis, along with neck, back, and shoulder pain [17,20,21]. These findings can potentially account for the trends noted in this study, specifically regarding the increased interest in terms of “hand pain” and “carpal tunnel” [16,17]. It is also possible that the rise in interest in the term “trigger finger,” is related to an increased usage of handheld devices [22]. However, there is currently a lack of evidence to support these conclusions and further research is needed to support these hypotheses.

In terms of active prevention of work-related hand and wrist dysfunction, other than limiting handheld device usage, it is possible that engaging in physical exercise may be of use, as it is correlated with a decreased incidence of CTS [16]. Myofascial stretching has been shown to be an effective treatment for CTS symptoms, making it plausible that it can be used for prevention as well and incorporated into the workday [23]. However, further studies should be conducted to see if this intervention has true preventative potential.

Other activity modifications during the lockdown

In a study conducted by Lund et al., it was suggested that occupations associated with high wrist angular velocities be targeted for preventative CTS interventions. Cleaners and laundry workers were noted to have some of the highest average wrist angular velocities. Gardeners and house painters were also recognized, although to a lesser degree [24]. The high wrist angular velocities seen in these occupations are related to the rapid, repetitive nature of wrist movements necessary to perform occupational-related tasks. During the lockdown, similar tasks performed by these occupational workers were adopted by the general public out of necessity (i.e., cleaning, laundry), while others pursued recreational activities (i.e., gardening, painting) [25-27].

Between the years of 2019 and 2021, consumer spending on cleaning products increased by 12% [28]. The repetitive wrist motions used while cleaning may have potentially led to hand dysfunction in some individuals, thus contributing to the upward trend of the search term “carpal tunnel syndrome” during the second and third quarters [29]. Additionally, a survey study from April 28, 2020, to May 12, 2020, in India, showed that 34.3% of participants reported an increase in physical load due to household chores during lockdown. The same sample reported that 45.8% of participants experienced an increase in neck and back pain [27]. While this study did not specifically inquire about hand pain, the utilized timeframe correlates with the significant upward trend of the search terms “hand pain” and “carpal tunnel syndrome” during the second quarter in India.

The pandemic’s influence on lifestyle did not cease with work and routine household and daily activities. Many individuals pursued other means of recreation during lockdown. Gardening and painting were among the many recreational projects that were favored, perhaps due to their compatibility with lockdown restrictions. In a separate analysis of Google Trends, India showed that an interest in gardening tended to peak when infection rates were high [25]. Additionally, from mid-2020 to mid-2021, Google reported a 250% increase in the searches for “DIY accent wall ideas” and “best interior paint” [26]. Since an increased risk of CTS is notable in both gardeners and painters, the adoption of these roles by non-professionals during the pandemic, as part of recreation, potentially contributed to the upward trend in the term “carpal tunnel syndrome” [9].

Limitations 

The greatest limitation of this study is that the chosen methodology can only support the existence of an association. While this study established an association between an increase of interest over time for the terms “hand pain,” “carpal tunnel,” and “trigger finger” and the increased prevalence of telework during this time period, it does not determine if the increased prevalence of telework was causative. In addition, our study only looked at trends within four English-speaking countries, providing a limited sample set.

According to SimilarWeb, Google is the top search engine used in the United States, United Kingdom, Canada, and India [30]. For this reason, trends from other search engines were not included in this study. However, by not investigating trends from other search engines, relevant data may have been missed.

Another limitation of this study is related to the methodology used by Google Trends. Since “interest over time” values are highly influenced by overall search volume, several factors could have confounded the results of our study. In addition, Google Trends does not allow users to access absolute values for a specific search term, making it difficult to assess whether or not there was a truly significant increase in trends.

## Conclusions

While the data in this study only identifies pre-existing associations, knowledge of these associations may be used to guide clinical practice. An assessment of patients’ prior knowledge and search strategies may influence the course of the patient interview and may necessitate that they be directed toward more reputable sources regarding their diagnoses.

Additionally, the associations identified in this study may be useful in guiding marketing strategies for healthcare systems and physician-owned groups. Based on the literature, certain occupations and activities may be associated with a higher incidence of hand pathologies, creating a more specific target audience for advertisement.
